# A Prediction Model of Incident Cardiovascular Disease in Patients with Sleep-Disordered Breathing

**DOI:** 10.3390/diagnostics11122212

**Published:** 2021-11-26

**Authors:** Jong-Uk Park, Erdenebayar Urtnasan, Sang-Ha Kim, Kyoung-Joung Lee

**Affiliations:** 1 Department of Artificial Intelligence, College of Medical Engineering, Konyang University, Daejeon 35365, Korea; jupark@konyang.ac.kr; 2 Artificial Intelligence Big Data Medical Center, Wonju College of Medicine, Yonsei University, Wonju 26426, Korea; edenbyra@yonsei.ac.kr; 3 Department of Internal Medicine, Wonju College of Medicine, Yonsei University, Wonju 26426, Korea; sanghakim@yonsei.ac.kr; 4 Department of Biomedical Engineering, College of Software and Digital Healthcare Convergence, Yonsei University, Wonju 26493, Korea

**Keywords:** sleep-disordered breathing (SDB), cardiovascular disease (CVD), artificial intelligence (AI), electrocardiogram (ECG), CVD risk factor

## Abstract

(1) Purpose: this study proposes a method of prediction of cardiovascular diseases (CVDs) that can develop within ten years in patients with sleep-disordered breathing (SDB). (2) Methods: For the design and evaluation of the algorithm, the Sleep Heart Health Study (SHHS) data from the 3367 participants were divided into a training set, validation set, and test set in the ratio of 5:3:2. From the data during a baseline period when patients did not have any CVD, we extracted 18 features from electrography (ECG) based on signal processing methods, 30 ECG features based on artificial intelligence (AI), ten clinical risk factors for CVD. We trained the model and evaluated it by using CVD outcomes result, monitored in follow-ups. The optimal feature vectors were selected through statistical analysis and support vector machine recursive feature elimination (SVM-RFE) of the extracted feature vectors. Features based on AI, a novel proposal from this study, showed excellent performance out of all selected feature vectors. In addition, new parameters based on AI were possibly meaningful predictors for CVD, when used in addition to the predictors for CVD that are already known. The selected features were used as inputs to the prediction model based on SVM for CVD, determining the development of CVD-free, coronary heart disease (CHD), heart failure (HF), or stroke within ten years. (3) Results: As a result, the respective recall and precision values were 82.9% and 87.5% for CVD-free; 71.9% and 63.8% for CVD; 57.2% and 55.4% for CHD; 52.6% and 40.8% for HF; 52.4% and 44.6% for stroke. The F1-score between CVD and CVD-free was 76.5%, and it was 59.1% in class four. (4) Conclusion: In conclusion, our results confirm the excellence of the prediction model for CVD in patients with SDB and verify the possibility of prediction within ten years of the CVDs that may occur in patients with SDB.

## 1. Introduction

Partial or total obstruction of the upper airway during sleep in sleep-disordered breathing (SDB) incurs respiration issues such as apnea or hypopnea as Figure S1 [1]. Approximately 2–4% of the world population suffer from SDB [2]. SDB is very common, with 3.2–4.5% of the people suffering from the disease [3]. An understanding of sleep apnea and hypopnea, although relatively common, has been poor in the past. However, it is getting a lot of attention nowadays because the prevalence of sleep apnea/hypopnea is rapidly increasing, associated with a recent increase in the obese population, and the complications are known to increase mortality rate [4]. The severity of sleep apnea-hypopnea syndrome is categorized by the apnea-hypopnea index (AHI) [5].

Recurrent sleep apnea or hypopnea acts as an acute stress factor in the cardiovascular system by inducing hypoxemia, reoxygenation, sudden pleural pressure changes, and awakenings of the central nervous system as Figure S2 [6]. Thus, SDB, untreated for a long time, increases the chance of development of CVDs such as hypertension, heart failure (HF), coronary heart disease (CHD), arrhythmias, and stroke, leading to mortality risk increase [7,8,9].

**Figure 1:**
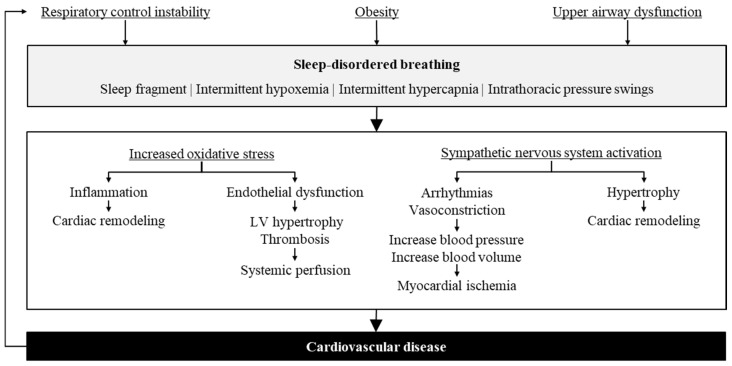
Pathophysiological consequence of sleep-disordered breathing and cardiovascular disease. LV = left ventricular.

Figure 1 shows the pathophysiology of SDB and CVD. In sleep apnea-hypopnea syndrome, the recurrent upper airway obstructions during sleep cause hypoxemia, reoxygenation, sudden pleural pressure changes, and the awakening of the central nervous system, which work as an abrupt cardiovascular stress factor [6]. Additionally, the sympathetic nervous system hyperactivity, selective activation of inflammatory pathways, vascular endothelial dysfunction, and metabolic dysregulation work as connection mechanisms for CVD [10].

The National Heart Lung and Blood Institute conducted a multi-center cohort study named the Sleep Heart Health Study (SHHS) to investigate the relationship between SDB and CVDs [11]. Participating in SHHS, Gottlieb et al. conducted prospective research to examine the relationship of SDB with CHD and arrhythmias. They found that the probabilities of the occurrence of CVD and arrhythmias were 68% and 58%, respectively, in patients with SDB, which are higher than those in healthy participants [12]. On the other hand, Redline et al. conducted a prospective study of SHHS to learn about the relationship between SDB and stroke. Patients with mild to severe SDB had a high affinity for developing ischemic stroke [13]. Other previous studies were concerning the predictors for CVDs, e.g., cholesterol, blood pressure, obesity, smoking, and electrocardiogram (ECG) [14,15,16]. Auer et al. found that ECG waves were related to CVD so that ECG abnormalities could be used as a predictor for CVD [16]. However, previous studies have investigated only for single disease target, or used group-wise analysis only such as morbidity and mortality rates. Additionally, the efficacy of the CVD predictors has not been evaluated for how well they can make actual predictions for the future development of CVD.

Therefore, in this study propose an algorithm based on artificial intelligence (AI) that can predict the development of CVDs, e.g., CHD, HF, and stroke within ten years, by using the ECG and common risk factors for CVD in patients with SDB. We believe that the potential for the prediction of comorbidities in patients with SDB, as verified in this study, will contribute to the realization of medical services.

## 2. Materials and Methods

### 2.1. Materials

SHHS is a cohort study conducted to investigate the outcomes of cardiovascular and sleep disorders [11]. It was a study confirming the SBD’s relationship with the risks of coronary artery disease, arrhythmias, and stroke that large hospitals in the United States participated in, as in in Figures S4 and S5. This cohort was approved by the Institutional Review Board in each participating center, and only the persons who signed the consent form were included as in the study. This study was conducted from 1995 to 2006 from 1 November 1995 to 31 January 1998, (baseline study), 6441 participants completed the survey about sleeping habits and health and went through polysomnography. Since they, they were monitored for the occurrence of CVD till April 2006.

**Figure 2:**
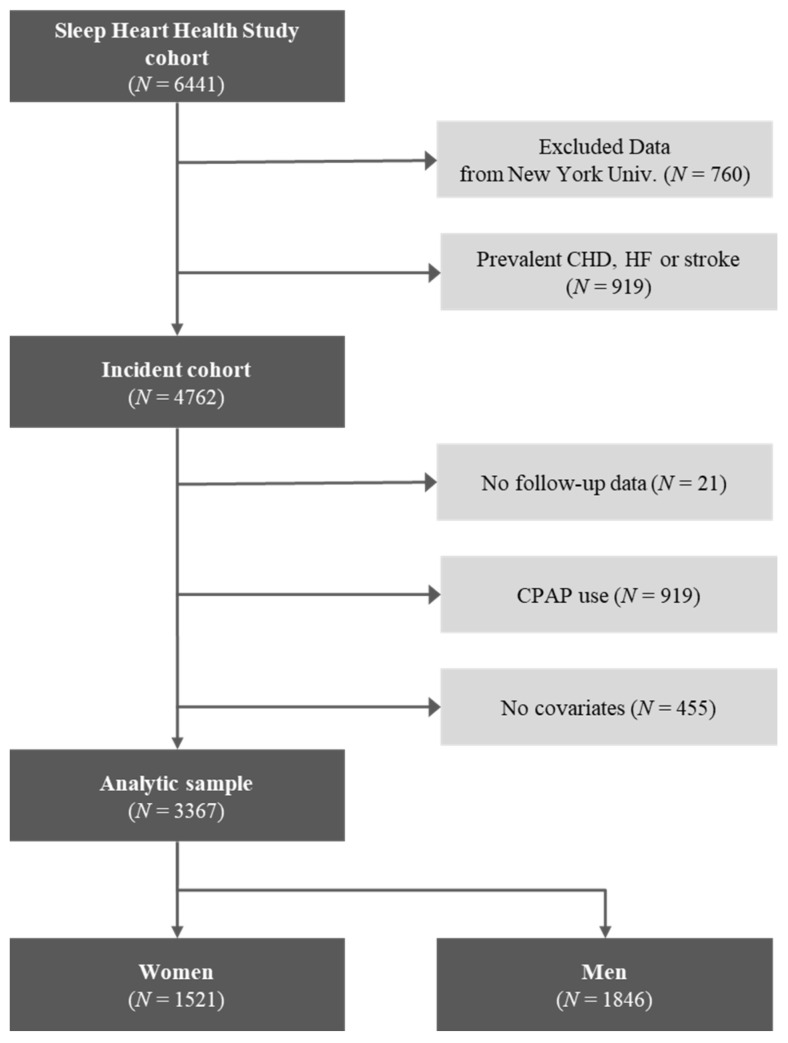
Ascertainment of the study sample. CPAP = continuous positive airway pressure.

Selected data in this study are displayed in Figure 2. For the design and evaluation of the algorithm, data from the 3367 research participants were divided into a training set, validation set, and test set in the ratio of 5:3:2. (Table S1). There was no significant difference in clinical characteristics between the training-validation set and the test set (*p* > 0.05).

### 2.2. Overview of CVD Prediction Model

**Figure 3:**
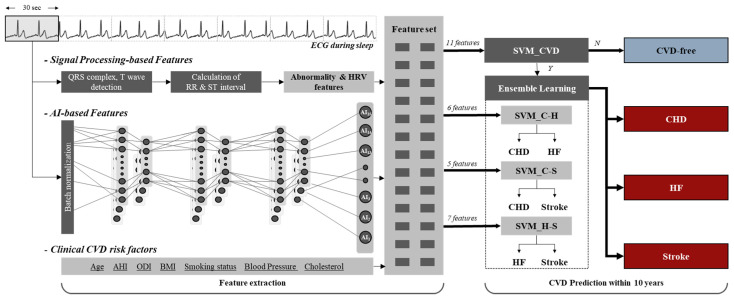
Prediction model of incident CVD outcomes in patients with SDB. SP = signal processing; AI = artificial intelligence; ECG = electrocardiogram; CVD = cardiovascular disease; SDB = sleep-disordered breathing; SVM = support vector machine; C = coronary heart disease; H = heart failure; S = stroke.

Figure 3 shows the model to predict the incident CVD outcomes in patients with SDB within ten years. To develop a prediction model for incident CVD (CHD, HF, and stroke), we extracted and selected a total of 23 feature vectors, i.e., 5 signal processing-based ECG features, 8 AI-based ECG features, and 10 clinical CVD risk factors. The selected features were used as inputs to the prediction model based on support vector machine (SVM), determining the development of CVD-free, CHD, HF, or stroke within ten years. First, when classified as CVD after passing through the SVM (SVM_CVD) that predicts CVD and CVD-free, the ensemble classifier is gone through to classify CHD, HF, and stroke. In this study, to classify three target classes, e.g., CHD, HF, and stroke, we predicted incident CVD outcomes using three SVM classifiers (SVM_C-H, SVM_C-S, and SVM_H_S).

### 2.3. Extraction of ECG Features

#### 2.3.1. Signal Processing-Based Features

For ECG signal, polysomnography data from baseline study were used, and the ECG signal was analyzed during sleep, from sleep onset to sleep offset. After detection of the QRS complex and T wave using the adaptive threshold algorithm and morphological method [17,18], QTc and STTc segments were calculated [19,20].

To calculate heart rate variability (HRV), we removed ectopic beats in the RR series, and this signal was defined as NN (normal-to-normal RR). For HRV analysis after interpolating the NN in equidistance, resampled it at 4Hz. The resample signal was transformed using the fast Fourier transform in 30 s segments, a square of which was the power spectrum density. Frequency bandwidths that were used to calculate the frequency domain characteristics were: very-low frequency (VLF: 0~0.04 Hz), low frequency (LF: 0.04~0.15 Hz), and high frequency (HF: 0.15~0.4 Hz). Finally, we extracted 18 signal processing-based ECG features after calculating the average and standard deviation of each feature during the whole sleep period (Table S2).

#### 2.3.2. AI-Based Features

Convolutional neural network (CNN) structure consisted of three convolutional layers with activation functions, each of which was followed by a max-pooling layer. A dropout technique was applied to the last three convolutional layers to avoid overfitting, as in the 1D CNN algorithm. The fully connected layer was used for final discrimination using a softmax activation function. Finally, the output was the likelihood that the observed data was produced by CVD or CVD-free event.

Finally, we extracted 30 AI-based ECG features after calculating the average and standard deviation, for the entire overnight sleeping period, of the AI-based features extracted from each node in the flattening layer of the CNN model during the whole sleep period (Table S3).

#### 2.3.3. Clinical Risk Factors

There have been ongoing studies about risk factors for CVD after the 2000s, so we referred to previous clinical literature to extract 10 CVD risk factors to use as inputs into the prediction model (Table S4).

### 2.4. Selection of Incident CVD Predictor

To select the optimal features for CVD classification, we confirmed for any significant difference between classes in the 58 features. For statistical analysis, depending on data type, two independent sample *t*-test and Chi-square tests were conducted for the training set, and we determined that each feature between classes was significantly different for *p*-value < 0.05 [21]. Additionally, to select the optimal feature out of the features with a significant difference, we applied support vector machine-recursive feature elimination (SVM-RFE) [22]. Through SVM-RFE, features were ranked in descending order of discernment, and combining the features in descending order of discernment, we conducted the learning and performance evaluation repeatedly. The above process was repeated four times to search for the optimal feature classifying CVD and CVD-free; CHD and HF; CHD and stroke; HF and stroke (Figure S3).

### 2.5. Prediction of Incident CVD Outcomes

After the process of feature extraction, we use the SVM model to predict the occurrence of CHD, HF, and stroke within ten years. First, when classified as CVD after passing through the SVM_CVD that predicts CVD and CVD-free, the OvO multiple class classifiers are gone through to classify CHD, HF, and stroke. OvO is the method of selecting a combination of two classes out of the K existing target classes and then selecting the class that acquired the most discrimination through K(K-1)/2 binary classifications [23]. In this study, to classify three target classes, e.g., CHD, HF, and stroke, we predicted incident CVD outcomes using three SVM classifiers (SVM_C-H, SVM_C-S, and SVM_H_S).

## 3. Results

### 3.1. Selection of Optimal Incident CVD Predictor

**Figure 4:**
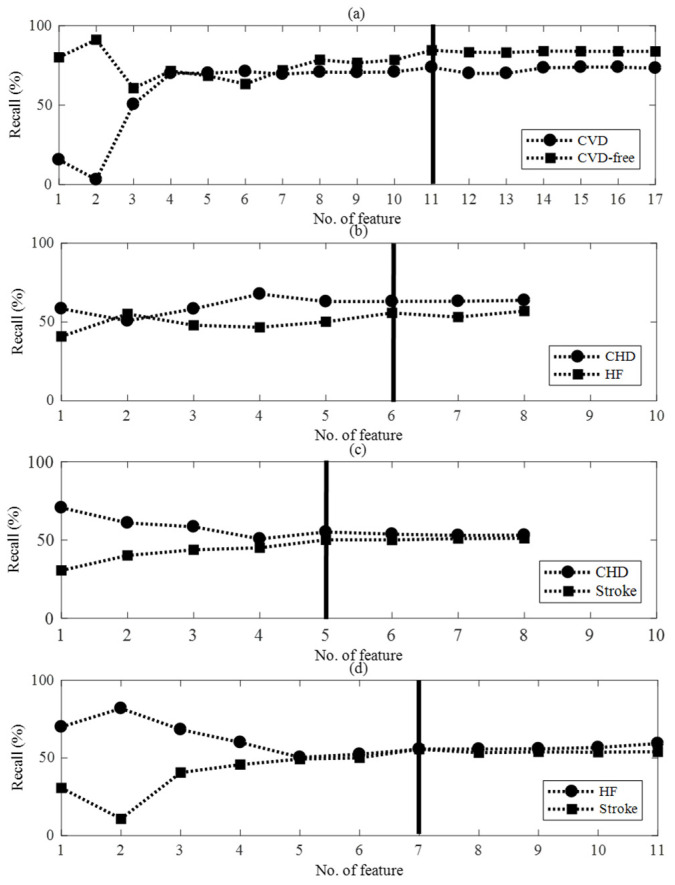
The classification performance according to number of features for feature selection. (**a**) CVD and CVD-free, (**b**) CHD and HF, (**c**) CHD and stroke, and (**d**) HF and stroke.

Figure 4 displays computed recalls after learning while adding one after another feature with a significant difference between classes in descending order of performance. We chose the feature showing the optimal performance with a minimal number of features, and 11, 6, 5, and 7 feature vectors were selected for SVM_CVD, SVM_C-H, SVM_C-S, and SVM_H-S. Out of the optimal features selected by incident CVD predictor, the AI-based ECG features going as inputs to the SVM_CVD, SVM_C-H, SVM_C-S, and SVM_H-S were three, two, one, and two, occupying a high rank. This shows a possibility that the AI-based novel parameter can be used as an essential factor predicting incident CVD, besides the common clinical CVD risk factor or ECG abnormality and HRV parameter (Table S5).

### 3.2. Performance Evaluation of Incidnet CVD Predictor

Table 1 and Table 2 show the results of incident CVD prediction for the training and test set, respectively. The recall and precision values for the entire training set regarding the CVD-free were 85.5% and 88.9%; CVD, 73.8% and 65.7%; CHD, 63.3% and 57.6%; HF, 55.2% and 45.0%; stroke, 52.2% and 47.8%, respectively, whereas the F1-score between CVD and CVD-free were 78.2% and in 4 class, 61.7%. For the entire test set, the recall and precision values of CVD-free were 82.9% and 87.5%; CVD, 71.9% and 63.8%; CHD, 57.2% and 55.4%; HF, 52.6% and 40.8%; stroke, 52.4% and 44.6% respectively, whereas the F1-score between CVD and CVD-free was 76.5% and in four classes.

## 4. Discussion

This study proposed a prediction method of CVD that occurs within ten years for patients with SDB. The purpose of our algorithm is to recognize the risks of SDB, assist active treatment of it, and prevent the CVD, which is a comorbidity of SDB.

In this study, we extracted a total of 58 feature vectors and selected the optimal feature vector through statistical analysis and SVM-RFE. Among the feature vectors selected with the optimal features, we confirmed the AI-based features that were proposed in this study showing excellent performance. In addition to the conventional CVD predictors, the new AI-based parameters showed a possibility to be used as a meaningful CVD predictor. Selected features were inputted into the SVM model to predict CVD, and four SVM models were designed for classifying CVD-free, CHD, HF, and stroke.

We evaluated the prediction performance of CVD and CVD-free in the test set depending on the severity of the SDB symptoms as well as gender. The F1-score was 73.9% in women, and 79.1% in men, so it could be confirmed that the prediction performed better in men than in women. Gottlieb et al. [9] and Redline et al. [10] analyzed the relationship between CVD and SDB according to the gender in the SHHS. As a result, it was confirmed that the relationship between CVD and SDB was higher for men than for women. The CVD predictor extracted from this study also showed a higher relationship for men than for women. Besides, the F1-score tends to fall with the severity of SDB for both men and women. This is seen to have been caused by the bias from low severity data set when learning, because those with AHI < 15 produced far more data than those with AHI >15 in the SHHS study. This performance is better than that of other models.

To learn about the difference between our proposed method and the conventional method, we experimented and presented the results in Table S6. We evaluated the performances by applying input features and AI models in various ways. For the AI model, we utilized the artificial neural network (ANN), convolutional neural network (CNN), support vector machine (SVM), linear discriminant analysis (LDA), and k-nearest neighbor (k-NN). We evaluated after dividing the input into the one that includes the feature extraction process and the one that does not. As a result, the proposed model in this study excelled over the other model in terms of performance. Through this, we developed a prediction model of an excellent performance using a small number of features that were acquired through the optimal feature selection process.

Therefore, we confirmed the excellence of the CVD prediction model for SDB, proposed in this study. We also presented a possibility of CVD prediction that may occur within the next ten years in the patient with SDB. Thus, we believe that the algorithm proposed in this study can be used to, recognize the risks of SDB, assist aggressive treatment, prevent the CVD, comorbidity of SDB.

In this study, we extracted the signal processing-based ECG features, AI-based ECG features, and clinical CVD risk factors. We also selected the optimal feature by using statistical analysis and SVM-RFE. Among the optima features, the AI-based ECG features were confirmed to have excellent performance in predicting the incident CVD outcomes. However, this study did not investigate how each feature is related to and CVD clinically. to confirm this, studies are needed that apply explainable AI technique to explain the meaning of each feature.

In this study, a method for predicting the occurrence of cardiovascular disease using a single electrocardiogram and major clinical indicators was presented. In addition, meaningful results were obtained by extracting new AI-based features. However, although classification of diseases is important, risk assessment for cardiovascular disease is more meaningful in clinical practice, and additional research is needed for this.

In patients with SDB and accompanying CVD, the mechanisms are increased oxidative stress and sympathetic nervous system activation [24]. For the prediction of the incident CVD, this study mainly dealt with ECG-based analysis, which is to check for ECG abnormality and changes in autonomic nervous system. However, in addition to ECG, it is necessary to analyze various signals simultaneously, such as oxygen saturation, which is associated with oxidative stress. We can expect the enhancement of performance and stability of a model by conducting research using various input signals and characteristics. In this study, we analyzed CVD such as CHD, HF, and stroke as targets. However, it is necessary to expand research about CVD outcomes by including more diverse details. Researchers have been interested in CVD risk factors for a long time. Thus, there have been many cohort research investigating the CVD risk factors [25]. Further, only SHHS data were used in this study. However, to verify our proposed method, extra data set should be additionally collected and analyzed.

## Figures and Tables

**Table 1:** The prediction results of incident CVD outcomes for the training set.

	Actual	CVD-Free	CHD	HF	Stroke	Precision (%)	
Predicted							
CVD-free		1620	87	45	71	88.9	
CHD		140	238	10	25	57.6	65.7
HF		52	31	85	21	45.0	
Stroke		106	20	14	128	47.8	
Recall (%)		84.5	63.3	55.2	52.2	^4^ F1: 61.7 ^2^ F1: 78.2	
			73.8				

CVD = cardiovascular disease; CHD = coronary heart disease; HF = heart failure; ^2^ F1 = F1-score for binary prediction (CVD-free|CVD); ^4^ F1 = F1-score for four class prediction (CVD-free|CHD|HF|stroke).

**Table 2:** The prediction results of incident CVD outcomes for the test set.

	Actual	CVD-Free	CHD	HF	Stroke	Precision (%)	
Predicted							
CVD-free		394	23	13	20	87.5	
CHD		37	56	2	6	55.4	63.8
HF		13	12	20	4	40.8	
Stroke		31	7	3	33	44.6	
Recall (%)		82.9	57.2	52.6	52.4	^4^ F1: 59.1 ^2^ F1: 76.5	
			71.9				

CVD = cardiovascular disease; CHD = coronary heart disease; HF = heart failure; ^2^ F1 = F1-score for binary prediction (CVD-free|CVD); ^4^ F1 = F1-score for four class prediction (CVD-free|CHD|HF|stroke).

## Data Availability

Not applicable.

## Supplementary Materials

The following are available online at https://www.mdpi.com/article/10.3390/diagnostics11122212/s1, Figure S1: Definition of sleep-disordered breathing, Figure S2: Pathogenesis of sleep -disordered breathing, Figure S3: Procedure of SVM-RFE for feature selection, Figure S4: Protocol of prospective cohort study, Figure S5: Participating Institutions of SHHS, Table S1: Subject characteristics of the SHHS dataset, Table S2: Description of 18 signal processing-based ECG features, Table S3: Description of 30 AI-based ECG features, Table S4: Description of 10 clinical CVD risk factors, Table S5: The results of feature selection for each classifier, Table S6: Comparison with other models.
