# Supplementary material for: A Prediction Model of Incident Cardiovascular Disease in Patients with Sleep-Disordered Breathing
Source: Diagnostics (Basel). 2021 Nov 26;11(12):2212. doi: 10.3390/diagnostics11122212 (PMC8700095; doi:10.3390/diagnostics11122212)
Supplement: Supplementary file 1 [file diagnostics-11-02212-s001.zip › diagnostics-1440075-supplementary.pdf]

diagnostics

MDPI

Supplementary Information for "A Prediction Model of Incident Cardiovascular Disease in Patients with Sleep-disordered Breathing"

Jong-Uk Park[1], Erdenebayar Urtnasan [2], Sangha Kim[3] and Kyoung-Joung Lee[4*]

[1] Department of Artificial Intelligent, College of Medical Engineering, Konyang University, Daejeon, Republic of Korea; jupark@konyang.ac.kr
[2] Artificial Intelligence Big Data Medical Center, Wonju College of Medicine, Yonsei University, Wonju, Republic of Korea; edenbyar@yonsei.ac.kr
[3] Department of Internal Medicine, Wonju College of Medicine, Yonsei University Wonju, Republic of Korea; sangha-kim@yonsei.ac.kr
[4] Department of Biomedical Engineering, College of Health Science, Yonsei University, Wonju, Republic of Korea; lkj5809@yonsei.ac.kr

***Corresponding Author: Kyoung-Joung Lee**

Address: (26496) 1 Yonseidae-gil Wonju-si Gangwon-do Republic of Korea

Tel: +82-33-760-2808, FAX +82-33-760-2852

E-mail: lkj5809@yonsei.ac.kr

Background

1. Definition of sleep-disordered breathing

Detection of SDB follows the standards by the American Academy of Sleep Medicine (AASM) that are displayed in Figure S1 [1,2]. Sleep apnea is the state of airflow obstruction for >10 seconds, while sleep hypopnea is defined as > 30% decrease in nasal pressure, 3% decrease in SpO2, or the occurrence of awakening [1]. Sleep apnea is categorized as obstructive sleep apnea (OSA) and central sleep apnea (CSA) depending on the mechanism of development [3]. In OSA, there is still a willingness for breathing, but in CSA, there is a lack of willingness for breathing [4].

An understanding of sleep apnea and hypopnea, although relatively common, has been poor in the past. However, it is getting a lot of attention nowadays because the prevalence of sleep apnea/hypopnea is rapidly increasing, associated with a recent increase in the obese population, and the complications are known to increase mortality rate [1].

The severity of sleep apnea–hypopnea syndrome is categorized by the apnea–hypopnea index (AHI) [5].

Apnea–hypopnea index (AHI): a total count of apnea or hypopnea per hour.

$$\text{AHI [1/hour]} = \frac{\text{Total number of apneas or hypopneas}}{\text{Total sleep time [hour]}} \quad (1)$$

- Mild : $5 \leq$ AHI < 15
- Moderate : $15 \leq$ AHI < 30
- Severe : AHI ≥ 30

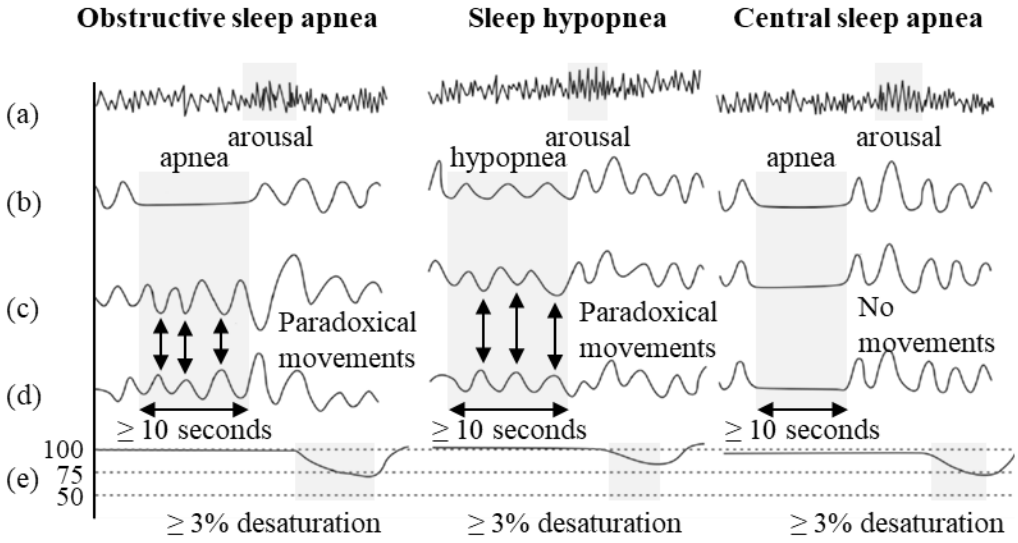

Figure S1 Definition of sleep-disordered breathing [18].

(A) electroencephalography (EEG), (b) airflow, (c) respiratory effort (chest), (d) respiratory effort (abdomen), and (e) oxygen saturation.

2. Pathogenesis of sleep-disordered breathing

Out of all mammals, sleep apnea–hypopnea syndrome is a disease appearing only in humans [6]. Anatomical changes in the human supralaryngeal vocal tract enabled the humans to speak languages, however, which, the structure was changed to collapse easily. Thus, it became predisposed to developing sleep apnea–hypopnea syndrome [6].

Notably, patients with sleep apnea–hypopnea syndrome, due to various predisposing factors, tend to show a narrower upper airway than those without, as shown in Figure S2 [7]. These structural characteristics increase airway closures, causing sleep apnea–hypopnea [7].

The goal of respiration is to maintain a constant concentration of blood gases, which is automatically regulated by the respiratory center located in medulla oblongata [8].

During sleep, activities in the respiratory center reduce, breathing patterns change, and responses to various external stimuli decrease [8]. Therefore, the respiratory center operates regularly, maintaining arterial blood gases at constants during awakening. Still, during sleep, hypoxia and hypercapnia can be induced by upper airway stenosis, decreased lung capacity, and decreased ventilation [7,9]. Such respiratory changes during sleep worsen in patients with airway obstruction and contribute to the mechanism of development of apnea or hypopnea repeatedly occurring during sleep in patients with SBD [9].

Sleep apnea or hypopnea is a phenomenon occurring only during sleep and is characterized as repeatedly occurring [10]. Figure S2 shows the mechanism of repeated awakening during sleep due to apnea and recurrent apnea development due to the awakening [7,9]. OSA develops due to anatomical factors in upper airway obstruction and decreased activity in the respiratory center during sleep. When apnea occurs, oxygen concentration decreases, and carbon dioxide increases in the blood. Awakening during sleep occurs if this state continues. Simultaneously with the awakening, the closed upper airway will open, and hyperventilation occurs to maintain normal concentration levels of blood gases. However, hyperventilation beyond the need will lead to the fall of blood carbon dioxide level below the standard, which will reduce the activity level in the respiratory center. Falling asleep in this state may lead to the occurrence of OSA or CSA. This malicious cycle becomes the causative factor of repetitions of sleep-awakening and respiration-apnea.

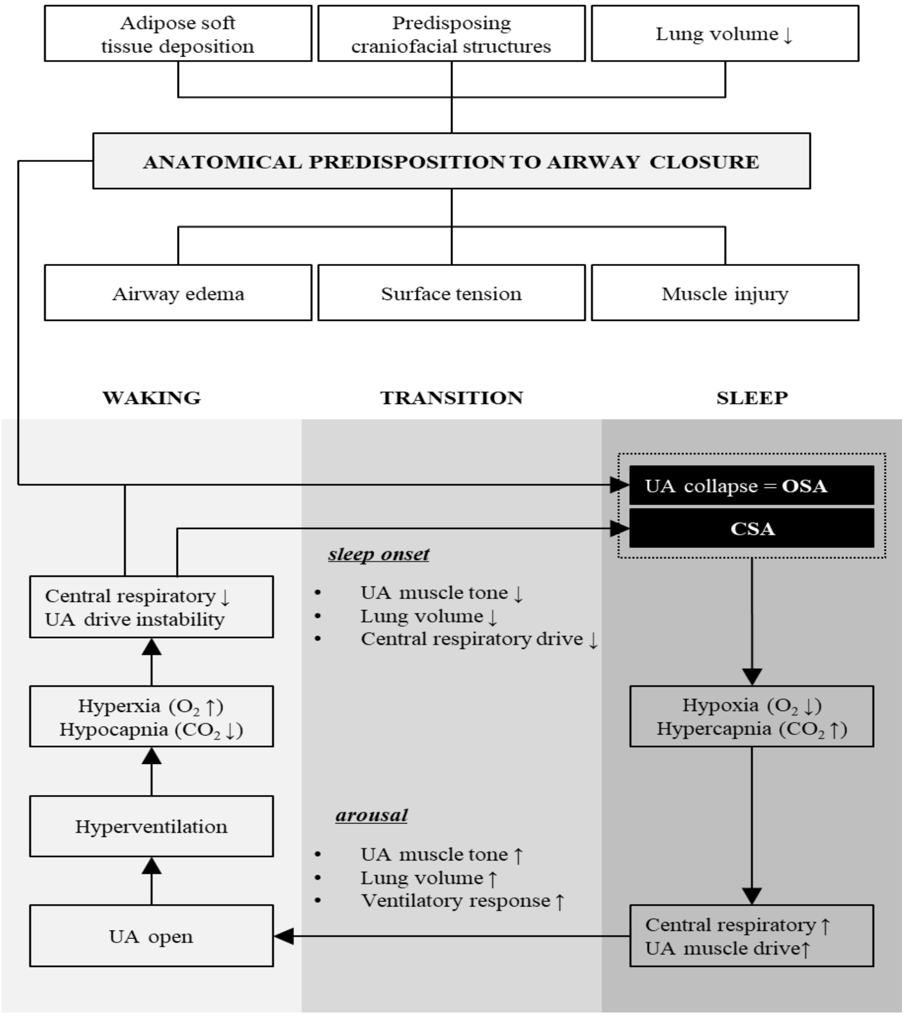

Figure S2 Pathogenesis of sleep -disordered breathing [18].

OSA = obstructive sleep apnea; CSA = central sleep apnea; UA: upper airway.

3. Convolutional neural network (CNN)

Convolution layer: A convolutional layer applies sliding filters to the input. To be specific, the layer convolves the input by moving the filters along the input vertically and horizontally and computing the dot product of the weights and the input and then adding a bias to them. The input to a convolutional layer is an m × m × r data where m is the height and width of the input data, and r is the number of channels. The convolutional layer will have k filters of size n × n × q, where n is smaller than the dimension of the input data and q, can either be the same as the number of channels r or smaller and may vary for each kernel. The size of the filters gives rise to the locally connected structure which is each convolved with the input data to produce k feature maps of size m−n+1.There are three main advantages of the convolution operation: the weight sharing, local connectivity, and invariance to the location [11].

Pooling layer: The pooling layer follows a convolutional layer and can be used to reduce the dimensions of feature maps and network parameters. The pooling layers are also in charge of down–sampling the spatial dimensions of the input. Max pooling is the most commonly used strategy, which divides the input data into a set of non-overlapping rectangles and outputs the maximum value of each such subsets [12].

Fully-connected layer: Fully-connected layers convert the feature maps into a 1D feature vector for further feature representation. Final discrimination of the input data is conducted at the fully-connected layer. At this stage, all the neurons are fully-connected, and the learning process is performed through feedforward and backpropagation algorithms [11]. Fully-connected layers perform like a traditional neural network and contain about 90% of the parameters in a CNN [13]. It enables us to feed forward the neural network into a vector with a predefined length.

Dropout: Dropout is a technique where randomly selected neurons are temporarily removed from the network, along with all its incoming and outgoing connections, during training. This technique has been shown to help in avoiding overfitting and preserve a network's ability to generalize [14].

Loss function: The loss function returns a value representing a penalty for incorrect classification. The goal then is to minimize the loss function when training. Mean square error (MSE) is a commonly used loss function in neural networks where equal value is placed on the error for each class. The calculation of MSE is defined in Equation 2.

$$MSE = \frac{1}{2n}\sum_i (y_i - \hat{y}_i)^2 \tag{2}$$

where $\hat{y}_i$ is the output of neural network, and y_i is label of target class.

4. Support vector machine (SVM)

SVM classifier aims at maximizing generalizing capability. It uses representative feature vectors of each class to search for the optimized decision hyperplane that has maximal margin between categories [15]. To find the optimal decision hyperplane that classifies each category, the distance from the separation boundary to the nearest point to (support vector) gets maximized, and the linear separation boundary can be defined as in Equation 3 [15]. Furthermore, the distance between the support vector and f(x) is $1/\|\omega\|$, and the object function having the optimal hyperplane that this distance becomes the greatest can be expressed as in Equation 5 under the condition in Equation 4 [15], where $1/2\|\omega\|^2$ is the term corresponding to the margin between the support vector and f(x); ξ is a slack variable allowing cognition error partially when linear separation is impossible, and; C determines the weight for a slack variable. Applying the Lagrange multiplier to Equation 5, one can define the optimal hyperplane function in specific space as in Equation 6 [15], where α_i^* is the Lagrange multiplier; (x, x_i) is Kernel function; y_i is the label of learning data; x is input data, and; x_i is a support vector.

$$f(x) = \omega^2 x + b \tag{3}$$

$$y_i(\omega^{\mathrm{T}} x + b) \geq 1 - \xi_i,\ \xi_i \geq 0, \mathrm{i} = 1,2,\cdots,N \tag{4}$$

$$\min J(\omega,\xi)=\frac{1}{2}\|\omega\|^2+C\sum_{i=1}^{N}\xi_i \quad (5)$$

$$f(x,\alpha^*)=\sum_{i=1}^{N}\alpha_i^* y_i K(x,x_i) \quad (6)$$

5. SVM-Recursive feature elimination (SVM-RFE)

SVM-RFE, a method that selects the optimal feature, measures the discriminatory power using the margin between classes that each feature vector possesses. With the assumption of a small margin between classes, the process of sequential backward elimination is conducted in descending order of discernment [16]. In other words, at first, discernment is measured using all feature vectors, and the feature with the least discernment gets removed. After this, using the features that are not removed, the feature removal process gets executed again, and this process repeats until only one feature is left. The last feature remaining is the feature with the biggest discernment, so choose the feature that shows maximal performance with a minimal number of features through the repeated learning and evaluation while combining the features in the order of discernment [17].

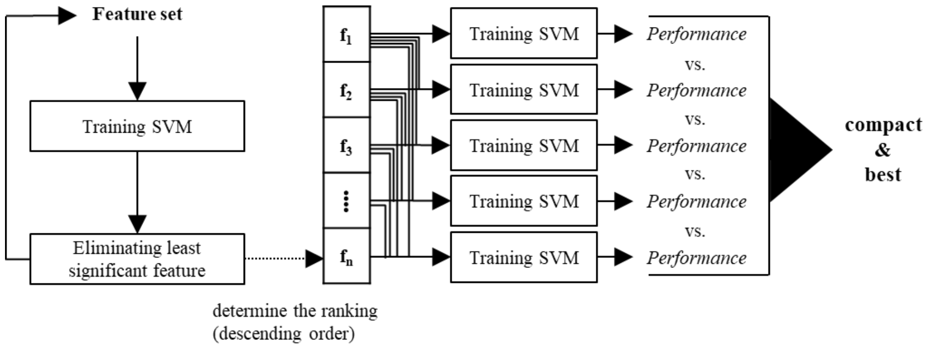

Figure S3 Procedure of SVM-RFE for feature selection [18].

Materials and Methods

1. Dataset

Table S1 Subject characteristics of the SHHS dataset.

	Training-Validation set	Test set	Total
No. of subjects	2,693	674	3,367
Age (year)	55.1±7.0	56.4±16.7	55.7±12.6
Gender (F/M)	1,216/1,476	305/370	1,521/1,846
BMI (kg/m2)	25.1±2.5	25.3±3.3	25±2.5
Smoking status (n)			
Current smokers	1257	323	1580

Former smokers	321	389	393
Never smokers	1028	363	1391
AHI (/h)	25.6±16.2	27.6±18.7	26.6±17.2
Systolic BP (mmHg)	125.2±8.9	126.1±11.4	124.4±10.7
Diastolic BP (mmHg)	72.4±5.6	72.3±5.2	72.3±5.1
Cholesterol (mg/dL)	207.1±6.2	205.5±6.0	206.4±6.3
CHD (n)	376	98	473
HF (n)	154	38	308
Stroke (n)	245	63	193

BMI = body mass index; AHI = apnea–hypopnea index; BP = blood pressure; CHD = coronary heart disease; HF = heart failure.

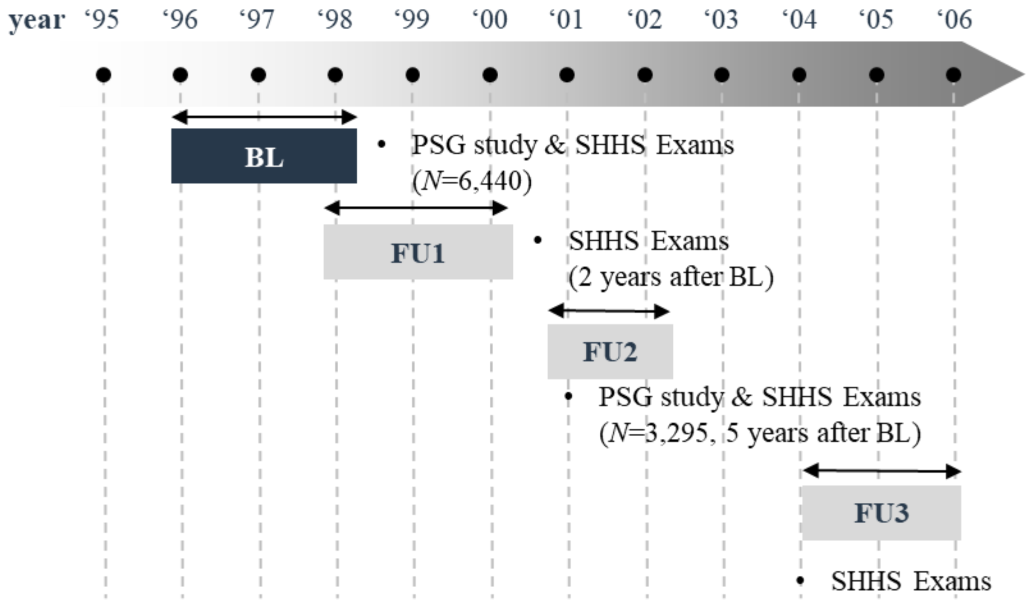

Figure S4 Protocol of prospective cohort study. BL = baseline study; FU = follow-up.

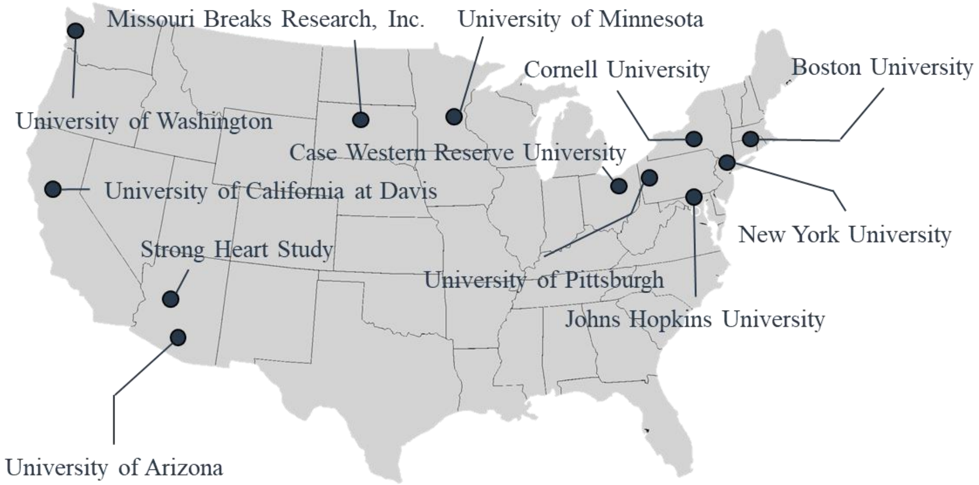

Figure S5 Participating Institutions of SHHS.

Results

1. Description of features

Table S2 Description of 18 signal processing-based ECG features.

Features	Description
ECG abnormality	
QTc_mean	the mean of corrected QT durations
QTc_SD	the standard deviation of corrected QT durations
STTc_mean	the mean of corrected ST-T segments
STTc_SD	the standard deviation of corrected ST-T segments
HRV features	
NN_mean	the mean of normal-to-normal interbeat intervals
NN_SD	the standard deviation normal-to-normal interbeat intervals
SDNN_mean	the mean of standard deviation of NN
SDNN_SD	the standard deviation of standard deviation of NN interval
RMSSD_mean	the mean of root mean square of the successive differences
RMSSD_SD	the mean of root mean square of the successive differences
P_{VLF}_mean	the mean of the power in very low frequency band
P_{VLF}_SD	the standard deviation of the power in very low frequency band
P_{LF}_mean	the mean of the power in low frequency band
P_{LF}_SD	the standard deviation of the power in low frequency band
P_{HF}_mean	the mean of the power in high frequency band
P_{HF}_SD	the standard deviation of the power in high frequency band
P_{LF}/P_{HF}_mean	the mean of the ratio P_{LF} and P_{HF}
P_{LF}/P_{HF}_SD	the standard deviation of the ratio P_{LF} and P_{HF}

Table S3 Description of 30 AI-based ECG features.

Features	Description
AI_1_mean	the mean of first node outputs in flatten layer
AI_1_SD	the standard deviation of first node outputs in flatten layer
AI_2_mean	the mean of second node outputs in flatten layer
AI_2_SD	the standard deviation of second node outputs in flatten layer
AI_{15}_SD	the standard deviation of 15th node outputs in flatten layer

Table S4 Description of 10 clinical CVD risk factors.

CVD risk factors	Type	CVD risk factors	Type
Age (year)	numerical	Systolic BP (mmHg)	numerical
Gender (F/M)	nominal	Diastolic BP (mmHg)	numerical
BMI (kg/m2)	numerical	Cholesterol (mg/dL)	numerical
Smoking status (F/C/N)	nominal	HDL cholesterol (mg/dL)	numerical
AHI(/h)	numerical	ODI(/h)	numerical

Table S5 The results of feature selection for each classifier.

Rank	SVM_CVD	SVM_C-H	SVM_C-S	SVM_H-S
1	**AI_{15}_mean**	**AI_4_mean**	**AI_{10}_mean**	**HDL cholesterol**
2	**ST-T_mean**	**HDL cholesterol**	**ST-T_mean**	**AI_3_mean**
3	**BMI**	**P_{LF}_mean**	**P_{LF}/P_{HF}_mean**	**P_{LF}_SD**
4	**Systolic BP**	**AI_{10}_mean**	**Diastolic BP**	**Cholesterol**
5	**AHI**	**P_{LF}/P_{HF}_SD**	**HDL cholesterol**	**AHI**
6	**AI_{11}_SD**	**AI_2_SD**	P_{LF}_mean	**AI_2_mean**
7	**P_{LF}/P_{HF}_mean**	P_{LF}_mean	BMI	**Systolic BP**
8	**ODI**	AHI	P_{LF}/P_{HF}_mean	P_{HF}_SD
9	**Smoking status**			AI_4_mean
10	**Age**			Smoking status
11	**AI_{12}_mean**			AI_6_SD
12	AI_1_mean			
13	AI_7_SD			
14	P_{LF}/P_{HF}_SD			
15	ST-T_SD			
16	P_{LF}_mean			
17	AI_3_mean			

SVM = support vector machine; CVD = cardiovascular disease; C = coronary heart disease; H: heart failure, S: stroke. **Bold**: selected optimal features. All features have significant difference between groups ($p<0.05$).

Table S6 shows the features that showed a statistically significant difference between classes as boxplots ($p<0.05$). The features that showed a significant difference between CVD and CVD-free; CHD and HF; CHD and stroke, and; HF and stroke were 17, 8, 8, and 11, respectively.

The features with a statistical difference were organized in the descending order of the best performance through SVM-RFE, as in Table S6. Besides, the repeat experiment was conducted and presented the finally selected feature in bold font.

2. Comparison performance with other models

Table S6 Comparison with other models.

Models	Input/features	^{2}F1	^{4}F1
ANN	ECG signals	69.7	53.1
ANN	SP and AI-based ECG features<br>CVD risk factors	74.0	57.7
CNN	ECG signals	70.9	52.3
CNN	SP and AI-based ECG features<br>CVD risk factors	74.8	58.0
SVM	SP-based ECG features<br>CVD risk factors	73.1	57.6
LDA	SP and AI-based ECG features<br>CVD risk factors	69.8	52.8
[Our study]	SP and AI-based ECG features<br>CVD risk factors	76.5	59.1

ANN = artificial neural network; CNN = convolutional neural network; SVM = support vector machine; LDA = linear discriminant analysis; SP = signal processing; AI = artificial intelligence; CVD = cardiovascular disease; ECG = electrocardiogram; ^{2}F1 = F1-score for binary prediction (CVD-free | CVD); ^{4}F1 = F1-score for four class prediction (CVD-free | CHD | HF | stroke).

To learn about the difference between our proposed method and the conventional method, we experimented and presented the results in Table S6. We evaluated the performances by applying input features and AI models in various ways. For the AI model, we utilized the artificial neural network (ANN), convolutional neural network (CNN), support vector machine (SVM), linear discriminant analysis (LDA), and k-nearest neighbor (k-NN). We evaluated after dividing the input into the one that includes the feature extraction process and the one that does not. As a result, the proposed model in this study excelled over the other model in terms of performance. Through this, we developed a prediction model of an excellent performance using a small number of features that were acquired through the optimal feature selection process. However, this study utilized SVM-RFE for feature selection. This may have resulted from extracting the features adequate for the SVM model in the feature selection process. Therefore, there is a need for conducting an additional experiment by using various feature selection methods [16].

REFERENCES

1. Berry, R.B. et al. The AASM manual for the scoring of sleep and associated events. Rules, Terminology and Technical Specifications. *Darien, Illinois, Am. Acad. Sleep Med.* **176**, (2012).
2. Sharma, Surendra K., Kriti Khanna, and Abhishek Sharma. "Sleep Disordered Breathing Disorders."
3. Yen, F.C. et al. A noninvasive technique for detecting obstructive and central sleep apnea. *IEEE Trans. Biomed. Eng.* **44**, 1262-1268 (1997).
4. Shin, C. Epidemiology and Definition of Sleep Disordered Breathing. *Tuberc. Respir. Dis.* **66**, 1-5 (2009).
5. Paquette, M, et al. Triglycerides, Hypertension, and Smoking Predict Cardiovascular Disease in Dysbetalipoproteinemia. *J. Clin. Lipidol.* **14**, 46-52 (2019).
6. Choi, S.J. Pathogenesis and Clinical Features of Sleep Breathing Disorder. *Tuberc. Respir. Dis.* **66**, 83-92 (2009).
7. Jordan, A.S. et al. Recent advances in understanding the pathogenesis of obstructive sleep apnea. *Curr. Opin. Pulm. Med.* **9**, 459-464 (2003).
8. Kim, W.S. Control of ventilation during sleep. *Sleep Med. Psychophysiology* **6**, 19-25 (1999).
9. Dempsey, J.A. et al. Pathophysiology of sleep apnea. *Physiol. Rev.* **90**, 47-112 (2010).
10. Yoon, D.W. et al. Mechanisms of obstructive sleep apnea development. *J. Kor. Sleep Soc.* **6**, 35-41 (2009).
11. Kiranyaz, S. et al. Real-time patient-specific ECG classification by 1-D convolutional neural networks. *IEEE Trans. Biomed. Eng.* **63**, 664-675 (2015).
12. Krizhevsky, A. et al. Imagenet classification with deep convolutional neural networks. *Adv. Neural Inf. Process. Syst.* **25**, 1097-1105 (2012).
13. Schwing, A.G. et al. Fully connected deep structured networks. *arXiv preprint arXiv*-1503 (2015).
14. Ba, J.L. et al. Adaptive dropout for training deep neural networks. University of Toronto (2014).
15. Duda, R.O. et al. Pattern classification. 2nd. Edition. *John Wiley & Sons* (2001).
16. Guyon, I. et al. Feature extraction: Foundations and applications *Springer* **207** (2006).
17. Koley, B.L. et al. Automatic detection of sleep apnea and hypopnea events from single channel measurement of respiration signal employing ensemble binary SVM classifiers. *Meas.* **46**, 2082-2092 (2013).
18. Park, J.U. Automatic classification of sleep apnea/hypopnea events using pulse oximeter. *Yonsei Uinversity* (2015)
